# Exploring Important Attributes, the Potential Use Cases and Feasibility of Introduction of Measles and Rubella Microarray Patches (MR-MAPs): Insights from Nine Countries

**DOI:** 10.3390/vaccines12091084

**Published:** 2024-09-23

**Authors:** Mateusz Hasso-Agopsowicz, Dijana Spasenoska, Maarten Paul Maria Jansen, Balcha Girma Masresha, Desiree Pastor, Abay Hagos Gebrekidan, Olivi Silalahi, Janice Woolford, Annet Kisakye, Anna-Lea Kahn, Birgitte Giersing

**Affiliations:** 1 Vaccine Product & Delivery Research, Department of Immunization, Vaccines & Biologicals, World Health Organization, 1211 Geneva, Switzerland; 2 Vaccine Preventable Diseases, World Health Organization Regional Office for Africa (AFRO/WHO), Harare P.O. Box CY 348, Zimbabwe; 3 Immunization Unit, Pan American Health Organization (PAHO), Washington, DC 20037, USA; 4 WHO Ethiopia Country Office, Addis Ababa P.O. Box 3069, Ethiopia; 5 WHO Indonesia Country Office, Jakarta 12940, Indonesia; 6 Family and Community Health-Immunization Focal Point, PAHO/WHO Guyana Country Office, Georgetown P.O. Box 10969, Guyana; 7 WHO Uganda Country Office, Kampala P.O. Box 24578, Uganda

**Keywords:** microarray patches, measles, rubella, vaccine

## Abstract

**Background**: Microarray patches (MAPs) are innovative, needle-free vaccine delivery systems, suitable for administration by minimally trained health care workers or trained community health workers. Their introduction may transform immunization programmes, particularly for vaccines where high coverage is required for population immunity, such as measles, and where vaccine delivery is challenging, such as in low- and middle-income countries. Recognizing the need to understand how best to tailor these products to reflect country priorities, workshops on measles and rubella MAPs (MR-MAPs) were conducted in multiple regions to collect insights on needs and preferences from relevant stakeholders at country level. **Methods**: The CAPACITI Innovation Framework was used to structure stakeholder discussions in nine countries in the period from August 2022 to July 2023. The discussions, building on the findings from a situation analysis on the barriers related to measles and rubella vaccine delivery, followed the four-step process outlined in the framework. **Results**: Key barriers hindering delivery of measles and rubella vaccines across the countries were in the categories of human resource management, service delivery, and demand generation. MR-MAP attributes that stakeholders believed would reduce or eliminate these barriers included ease of preparation and administration, improved thermostability, fewer (ancillary) components, and single-dose presentation. Some attributes such as the site of administration, wear time, and storage volume could exacerbate certain barriers. Based on an understanding of key barriers, product attributes, and underserved populations, stakeholders identified several potential use cases for MR-MAPs: (i) delivery at a fixed health post, (ii) delivery through outreach sessions conducted by health workers, and (iii) administration by community health workers. To enable robust national decision making about the introduction of MR-MAPs and successful implementation, global and national evidence on feasibility and acceptability of MR-MAPs should be generated. To prepare for the potential introduction of MR-MAPs, immunization programmes should evaluate their immunization policies based on their preferred use cases and modify them if needed, for example, to enable community health workers to administer vaccines, along with making programmatic adjustments to waste management and training. **Conclusions**: MR-MAPs have the potential to reduce key barriers to MR delivery. Yet, their future impact depends on the ability of global stakeholders to steer the development of MR-MAPs to be responsive to country needs and preferences. The generation of evidence to enable robust decision making, timely modification of vaccine policies, and addressing programmatic considerations will be key to successful uptake.

## 1. Introduction

Microarray patches (MAPs) are anticipated to become a transformative, needle-free vaccine delivery innovation. Comprised of micro-projections on a ‘patch’ laden with a dried vaccine, these patches, when applied to the skin, directly deliver the vaccine into the dermis and/or epidermis. MAPs remove the need for vaccine reconstitution, thereby improving safety during administration, enabling administration by minimally trained personnel, and removing sharps waste. They also appear to be less painful than injectable vaccines which could increase the willingness to be vaccinated among those for whom fear of needles or pain is a significant issue. Currently, MAPs are under development for various vaccines, including measles and rubella (MR) vaccines. The latter receive a significant focus due to the need for high coverage to establish and maintain herd immunity and achieve the global goal of eliminating MR. Yet, 22 million children in 2022 did not receive their first dose of measles containing vaccine, and the number of measles cases increased to 9.2 million [1]. Hence, innovations in delivering vaccines are needed to improve access, increase vaccination coverage, and reduce inequity. MR-MAPs have been identified by WHO’s Strategic Advisory Groups of Experts on Immunization (SAGE) as a priority innovation to help eliminate measles and rubella [2].

The single-dose format of MR-MAPs eliminates the hesitancy to open multi-dose vials when only a few children are present at the vaccination session. This reduces missed opportunities for vaccination (MOVs) and vaccine wastage. Importantly, MAPs could also offer improved thermostability, which could reduce the need for cold chain during vaccine delivery in outreach or campaigns [3]. Given these operational advantages, MAPs have the potential to increase equitable coverage and facilitate vaccine administration in inaccessible areas, playing a pivotal role in reducing the disease burden [4]. MAPs were prioritized by the Gavi, World Health Organization (WHO), and UNICEF-led Vaccine Innovation Prioritisation Strategy (VIPS) in 2020 to advance development of vaccine innovations with expected impact on equity and coverage [5].

Significant work has been conducted to advance MR-MAP development. Examples include development of a target product profile for MR-MAPs [6]; identification and validation of use cases [7]; acceptability and human factor studies [8,9]; evaluation of thermostability [10,11]; impact and cost-effectiveness study [4]; and estimation of MR-MAP demand [12]. A phase I/II trial in nine-month-old infants in the Gambia has shown that safety and immunogenicity of MR vaccines delivered by a MAP is comparable to MR vaccines delivered by needle and syringe (N/S) [13]. Despite these efforts, it is unlikely that MR-MAPs will become available before 2029. Broad-scale and early availability requires substantial at-risk investment in manufacturing for MAP developers, MR manufacturers, and partners, as there are uncertainties in MR-MAP demand [14,15].

Historical trends have shown that a lack of understanding regarding the specific needs and preferences of low- and middle-income countries (LMICs) often culminates in vaccine products that are ill suited for these settings [16]. In the context of MR-MAPs, it is paramount to gain a clear understanding of countries’ viewpoints on several issues: the potential use cases of MR-MAPs, important MAP attributes for priority use cases, and the evidence countries deem essential to confidently decide on the introduction of MR-MAPs. Gaining insights into these issues is instrumental to understand the demand for MR-MAPs, guide investments in their production, ensure the developed products are tailored to countries’ requirements, and facilitate their rapid uptake into national immunization programmes. Given the ongoing investments in manufacturing facility, these questions need to be answered now to influence the finalization of the MAP design. Furthermore, the insights will chart the course for decision-making pathways, ensuring that the right evidence to enable robust decision making is collected during development, and appropriate guidance is on hand to aid countries in adopting and using MR-MAPs [3].

Recognizing the importance of country-level perspectives to evaluate the product innovation, we have conducted consultations with national stakeholders from nine countries to (1) determine and prioritize immunization barriers specific to MR vaccines which can be impacted by changing the way vaccines are delivered; (2) identify critical attributes of MR-MAPs to reduce or eliminate these prioritized barriers; (3) understand and validate potential use cases for MR-MAPs; (4) identify likely data and evidence needed for the eventual introduction of MR-MAPs before they are approved for use, but not yet deciding whether to introduce the product; (5) familiarize stakeholders with MR-MAPs, their attributes, and status of development. In this paper we report the findings from these consultations.

## 2. Methodology

### 2.1. The CAPACITI Innovation Framework

**Figure 1:**
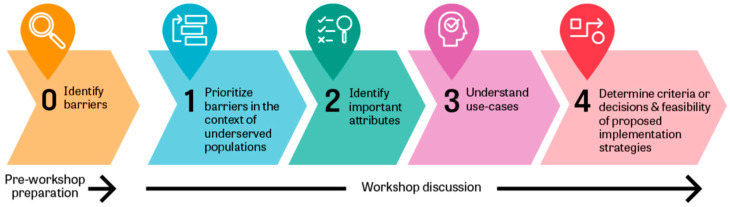
The methodology of the Innovation Framework workshops.

The CAPACITI Innovation Framework, developed by WHO, was used as a methodology for conducting the workshops. The CAPACITI Innovation Framework is a tailored process, which facilitates discussion, deliberation, and communication between stakeholders from diverse levels and disciplines within the immunization system to agree on potential programmatic implications of an innovation. Furthermore, it guides discussions around perceived value and acceptability requirements of the innovation in comparison with the current practice and seeks to identify important decision criteria and the evidence needed for eventual robust decisions on the introduction of the innovation. The Innovation Framework is available in an Excel workbook, which has been adapted with specific questions for MR-MAPs. The outcomes of each of the workshops have been systematically documented in separate, dedicated workbooks. Figure 1 shows the steps of the framework, which are further described below. A full methodology of the CAPACITI Innovation Workshops is described elsewhere [17].

### 2.2. Step 0: Identifying Barriers

To understand the country context, a comprehensive evidence-based situation analysis of the national immunization programme performance was conducted [18]. Existing documents on immunization programme performance were reviewed and evidence around barriers was documented, for each of the seven EPI categories: programme management; human resources management; vaccine supply, quality and logistics; service delivery; immunization coverage and adverse events following immunization (AEFI); disease surveillance; and demand generation. The outcome of the situation analysis was a list of programmatic barriers undermining high and equitable vaccine coverage. The list of identified barriers was validated by national stakeholders.

### 2.3. Step 1: Prioritizing Barriers in the Context of Underserved Populations

The list of programmatic barriers to equitable and high vaccination coverage was reduced to barriers relevant to MR vaccine coverage and equity, through a discussion with participants. The relevance of barriers and their impact on coverage of MR vaccines varies at sub-national level depending on the context of the underserved and unvaccinated populations. Thus, each country defined their own specific sub-national settings based on specific characteristics (e.g., geography, socio-economic, and cultural context). For instance, in Ethiopia, the stakeholders specified agrarian, pastoralist, and urban settings, while in Indonesia, the settings were grouped based on their socio-economic development. In Uganda, the settings reflected the diverse terrains and communities–mountainous regions, conflict-prone areas and islands, inhabited by nomadic and fishing communities, as well as refugees and displaced populations. Stakeholders discussed a comprehensive list of barriers within the identified settings and determined the highest priority barriers for each. They then finalized a combined list of the highest priority barriers by automatically including those that are cross-cutting across settings and debating the inclusion of additional high-priority barriers relevant only to specific underserved populations.

### 2.4. Step 2: Identifying Important Attributes of MR-MAPs

For each of the high-priority barriers identified in Step 1, stakeholders were asked to evaluate whether MR-MAP attributes, identified in the WHO/UNICEF Target Product Profile (TPP) for MR-MAPs [6], could have an effect on the barrier. An attribute could reduce or eliminate the barrier, increase the barrier, have an indirect benefit, impose indirect challenges, or it might not have any effect. More than one attribute and effect could be selected per barrier.

### 2.5. Step 3: Understanding the Potential Use of MR-MAPs

To effectively guide MR-MAP product development, it is essential to anticipate how this innovation would be used by immunization programmes. Identifying the likely use cases will further validate the importance of critical product attributes, help to understand the feasibility and acceptability of MR-MAPs, and inform introduction strategies. Prior studies have explored MR-MAPs’ use based on the service provider (health worker, community health worker, or self-administration) and delivery location (fixed health post, outreach, other setting) (Supplementary Figure S1) [7]. The six distinct use cases that emerged from the prior studies were consolidated into four use cases considered during the workshops. Participants discussed whether these use cases could be feasible and desirable in their country and reflected appropriate and desired applications of MR-MAPs to reduce the identified barriers.

### 2.6. Step 4: Understanding Decision Pathways and Decision Criteria for Decision Making About the Introduction of MR-MAPs in the Immunization Programme at the National Level

To understand the introduction decision pathway at the national level, stakeholders were asked to describe the steps required for decision making about the potential introduction of MR-MAPs in the immunization programme.

Focusing on the potential decision to introduce MR-MAPs, stakeholders were asked to agree on important decision-making criteria and key evidence needed to ensure robust decision making, drawing on their previous discussions throughout the workshop. The design of this group discussion was informed by the CAPACITI decision-support tool guidance [19], in particular, regarding part 2 identifying decision criteria, and part 3 evidence assessment, which provides guidance on the development of an evidence assessment plan. Finally, stakeholders were asked about the perceived feasibility of delivering two MR vaccine presentations in a country: a mixed delivery of MAP and needle and syringe (N/S).

### 2.7. Selection of Countries for Engagement

To capture multiple and varied national contexts, a long list of target countries was developed. Key considerations for choosing the countries included geographic diversity, number of zero-dose children, sub-national variation in vaccination coverage, and unique features (e.g., local production of vaccines, large number of special populations such as refugees). Focal points in the identified countries were contacted through official communication channels by WHO and partner organizations. Following initial discussions around the feasibility of conducting such workshop (e.g., elections preventing workshops to take place, stakeholders busy responding to an outbreak or conducting a vaccination campaign), and in consultation with WHO Regional Offices, nine countries were identified.

Between August 2022 and July 2023, four workshops were conducted with a total of nine countries, and three WHO regions, through single-country and multi-country engagements. Single country workshops were conducted in Ethiopia, Indonesia, and Uganda, while Guyana, Belize, Trinidad, and Tobago, Suriname, Jamaica, and Barbados were engaged in a multi-country workshop.

### 2.8. Selection of Workshop Stakeholders

The list of participants for each workshop was developed in consultation with the respective Ministry of Health, WHO headquarters, Regional Offices, or Country Offices. The general guidance for each country was to nominate 30 to 50 participants who are relevant to the measles and rubella vaccination in the country. They were encouraged to include a wide range of stakeholders, for instance various departments within the ministry, sub-national focal points, health workers, national immunization technical advisory groups, academia, implementing partners, and civil society organizations. The list of organizations represented varied between countries, although the general profile was within the suggested guidelines. The details of the workshop logistics are listed in the Supplementary Material Table S1.

## 3. Results and Discussion

### 3.1. Step 1: Identification and Prioritisation of Barriers to Measles and Rubella Immunization

In each country, the situation analysis revealed 30-40 barriers of the national immunization programme performance across all seven EPI categories. The list was narrowed to 15–18 barriers through a prioritization discussion during the workshop. Some of the prioritized barriers hindering MR vaccination coverage and equity included an inadequate supply of health staff coupled with a high turn-over of staff, and inadequate training. Furthermore, the transport of vaccines to remote delivery settings was a big challenge, together with inadequate cold chain quality and inappropriate monitoring of heat or freeze exposure during transport. The logistics were further challenged by long distance and travel time, and the need for intensified outreach to special and remote populations. Challenges around data quality and accuracy of denominators for calculating target populations were prevalent across the majority of settings. Lastly, demand-related barriers were prevalent such as fear of multiple injections, cultural and religious concerns, and lack of confidence and trust in vaccines. The full list of prioritized barriers is available in the Supplementary Material.

### 3.2. Step 2: Identification of Important Attributes of MR-MAPs

**Figure 2:**
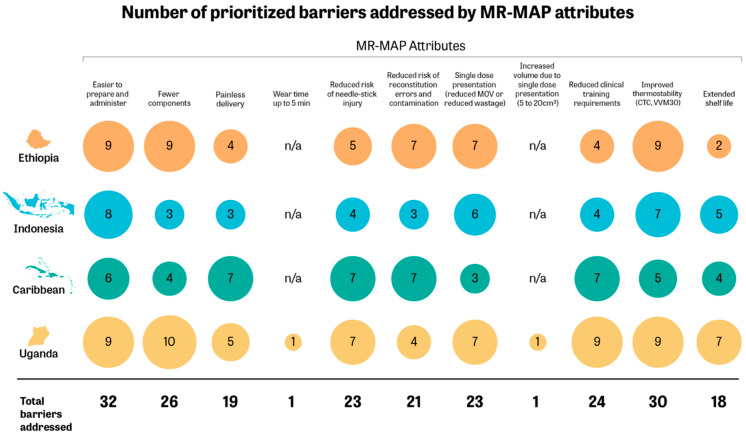
Number of prioritized barriers addressed by MR-MAP attributes. CTC (controlled temperature chain), VVM30 (vaccine vial monitor 30), MOV (missed opportunities for vaccination), n/a (not applicable).

Most of the positive effects of MR-MAPs on barriers were associated with ease of preparation and administration, single-dose presentation, improved thermostability, and fewer ancillary components required for vaccination (Figure 2). There is variability across countries in the number of barriers that stakeholders believed a specific attribute could impact, and there are differences across countries regarding which attributes were perceived as having the potential to impact the widest range of barriers. For instance, in Ethiopia, the attribute around reduced clinical training requirements could address only four barriers, while in Uganda, it could positively impact nine barriers. Moreover, there were some attributes that could negatively affect barriers, such as the site of administration, the required wear time of up to five minutes, and the relatively bulkier volume of MR-MAPs as compared to a single MR dose within a multi-dose vial. The nuanced discussions about the impact of specific MR-MAP attributes on the immunization barriers are further explained below.

#### 3.2.1. Focus on Specific Attributes: Thermostability

Improved thermostability of MR-MAPs is seen as a significant leap towards ensuring broader vaccine access, especially in areas where maintaining a cold chain is difficult. This can particularly aid in reaching vulnerable or hard-to-reach populations. The WHO/UNICEF TPP for MR-MAPs stipulates that vaccine stability should surpass that of the current MR vaccines. MAPs should have vaccine vial monitors (VVMs)—small temperature-sensitive indicators that gradually darken as the vaccine is exposed to cumulative heat. The range of acceptable VVMs consists of those with a 14-day maximum cumulative heat exposure (VVM14), and ideally those with a 30-day threshold (VVM30) or more, allowing for greater and longer exposures to temperatures beyond the standard 2 to 8 °C cold chain. MR-MAPs should also be compatible with use in a controlled temperature chain (CTC), tolerating a single excursion out of the standard cold chain of at least three days at 40 °C [6].

The positive impact of potentially enhanced thermostability resonated with all national immunization programme stakeholders. This attribute could reduce logistical challenges in transporting vaccines over long distances to remote sites, reaching currently underserved populations. Moreover, it could reduce the risk of closed vial wastage due to heat or freeze damage, which may occur with inadequate maintenance of the cold chain or mishandling of the vaccine, challenges that are currently not well documented.

However, it is essential to also recognize the new challenges MR-MAPs might introduce. Implementing vaccine delivery under CTC conditions would necessitate initial specialized training. Furthermore, concerns were raised about potential wastage; poor microplanning could lead to an excessive number of vaccines being taken out of the cold chain into CTC and as they cannot then be returned to the cold chain, they must be discarded if they are unused at the end of the specified number of days. This is a particularly important consideration in the context of identified challenges around accurate estimates of the size of populations who need to be vaccinated.

#### 3.2.2. Focus on Specific Attributes: Wear Time

The WHO/UNICEF TPP states that the acceptable wear time for MR-MAPs is up to five minutes and optimally less than one minute. A MAP should be worn for the specified time, observed and removed by an HCW, trained lay health worker, or caregiver [6]. Among workshop stakeholders and those familiar with MAPs, there was a shared concern that a duration up to five minutes might extend the immunization sessions.

However, stakeholders in the workshops argued that with some adjustments, the five-minute wear time could be accommodated into routine immunization sessions. Generally, caregivers spend about 30 min at vaccination points for the monitoring of AEFIs. Additionally, the time spent in a clinic often exceeds an hour, to include activities such as health measurements, vitamin administration, and informational discussions about vaccines, nutrition, and more. As such, accommodating a five-minute wear time during standard immunization sessions appears feasible. The challenges might differ for immunization campaigns or house-to-house delivery, where the five-minute wear time could exceed the length of a contact point during vaccine delivery. The challenges associated with the five-minute wear time could potentially be reduced by planning the order of administered vaccines—starting with the MR-MAP and then proceeding with other injectable vaccines.

Stakeholders expressed the need for additional data on the consequences of wearing the MAP for too long, and on the protocol to supervise a child and ensure that the MAP stays at the site of administration. They emphasized that if a MAP needs to be held in place for the duration of wear time, it would be preferred for caregivers to do so rather than health workers, to avoid increasing the time burden and reducing session quality.

In conclusion, there is preference for wear time of up to one minute over five minutes, and caregivers to hold the child’s body part still during application and wear time over health workers.

#### 3.2.3. Focus on Specific Attributes: Site of Administration

The WHO/UNICEF TPP specifies that the site of application should not impede efficacy of vaccination, however, it does not specify the exact site. The noted preferable locations on infants and toddlers are those that are less likely to be disturbed and/or removed (such as the scapula) or the upper arm in older children. However, some of the MAPs in development are being tested on other anatomical sites such as the wrist. Hence, during the discussions in Uganda, the feasibility and desirability of the wrist as a delivery site was explored.

Stakeholders expressed preference for the flexibility to apply the MR-MAP at various anatomical sites. There were some concerns around the wrist as a delivery site. Some argued that children could remove the patch, while others proposed that with robust adhesion, this issue could be mitigated. In other countries, marking of the wrist might have some negative cultural and religious connotation, so appropriate communication strategies must be in place to avoid significant challenges around community acceptance. However, despite some reservations, participants agreed that the delivery site is not a critical concern that would significantly impede introduction of MR-MAPs.

Furthermore, participants expressed a desire for more evidence on how different skin factors, such as moisture levels, thickness, and external factors like air humidity, could affect effectiveness of MR-MAPs.

#### 3.2.4. Focus on Specific Attributes: Increased Volume

The minimal acceptable target for MR-MAPs cold chain storage volume per dose is specified as no greater than a single-dose vial of injectable MR vaccine (21.09 cm^3^). Yet, given that the current practice is the use of a five or ten multi-dose vial, with volumes ranging between 5.5 cm^3^/dose and 2.1 cm^3^/dose in secondary packaging, the single-dose presentation of MR-MAPs would inevitably lead to increased cold chain volume, bringing a set of direct and indirect challenges. Directly, the larger volume could result in larger cold chain space requirements prior to the final stages of vaccine delivery, thereby putting a strain on existing infrastructure, especially in regions where cold chain systems might already be at capacity. Indirectly, this increased volume implies a surge in needed financial resources for the necessary cold chain enhancements and other logistical considerations. However, the impact of MAPs on cold chain requires further evaluation as it depends on the final MAP attributes, available cold chain equipment, and capacity in countries.

While the MR-MAPs could offer thermostability benefits, these might not be enough to entirely counterbalance the cold chain constraints associated with their increased volume. The CTC advantage, though significant, is only available as an option during the final stages of vaccine delivery, also referred to as the “last mile”, and it typically applies for only a few days just prior to administration.

Given these concerns, participants specified that before any widespread introduction of MR-MAPs, a thorough assessment of their impact on existing cold chain systems would be needed. Such evaluations could be conducted before or integrated into pilot studies, ensuring that countries are adequately prepared to handle the logistical demands of these patches.

#### 3.2.5. Acceptability of MR-MAPs

The introduction of MR-MAPs into immunization programmes may impact vaccine acceptance. It could alleviate barriers associated with fear or dislike of multiple injections during a single visit, often a concern for both caregivers and health workers. The trust in the immunization process could be further bolstered if community health workers or caregivers were trained to administer these patches, increasing ownership of the vaccination process, and reducing distrust in the health system. Additionally, workshop participants acknowledged that by eliminating vaccine reconstitution errors, MR-MAPs can reduce the occurrence of adverse effects post-immunization, alleviating concerns that caregivers might have around vaccine safety.

However, the novel vaccine presentation might also exacerbate some demand barriers. If communication and community sensitization prior to introduction of MR-MAPs is inadequate, hesitancy might be observed. Doubts might arise around the novel presentation of the vaccine both among caregivers and health workers, as it is different from the current practice a needle and syringe.

### 3.3. Step 3: Understanding the Potential Use Cases of MR-MAPs

**Figure 3:**
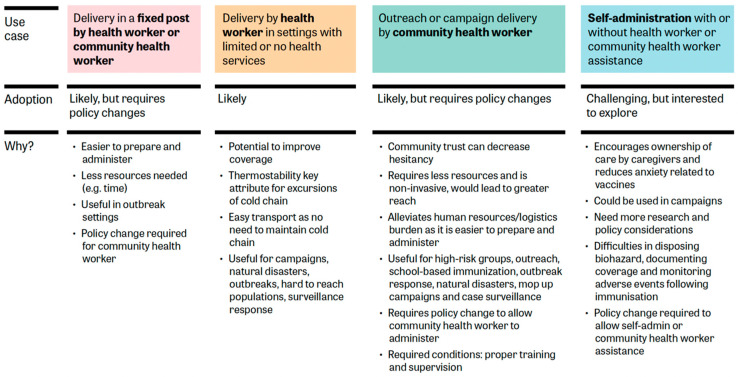
Use cases to understand the potential use of MR-MAPs. HW, health worker; CHW, community health worker; SIAs, supplementary immunization activities; MR-MAP, measles and rubella microarray patch.

All use cases discussed during the workshops, and their anticipated adoption and rationale, are shown in Figure 3. Delivery of MR-MAPs at a *fixed post by health or community health workers*, where fixed health post is defined as a permanent structure which has full cold chain capabilities, was deemed attractive for stakeholders from across the participating countries. MR-MAPs, with their ease of administration and potential to reduce injection fear, can benefit immunization programmes by easing the task of vaccination and reducing training needs. For delivery of MR-MAPs *by health workers in settings with limited or no health services* (including delivery in areas that do not have access to fixed health posts and have reduced or no cold chain capacities), workshop participants attributed the value of MR-MAPs to their improved thermostability, making the devices apt for challenging terrains and able to improve reach, which could augment MR vaccine coverage. Provided sufficient CTC time allowances and appropriate demand forecasting, the flexibility afforded by removing cold chain constraints was cited as making MR-MAPs a desirable option not only for routine vaccination but also for targeted campaigns and situations like humanitarian disasters and outbreaks catering to remote populations.

The use of MR-MAPs in *outreach or campaign delivery by community health care workers* was perceived as highly favourable by workshop participants. The foundation of this preference is the belief that vaccinations offered by community health workers operating in settings where they are well known and respected can elevate trust in vaccines, fostering higher acceptance. Deploying MR-MAPs through these workers was viewed as resource efficient, promising a more extensive outreach strategy. Additionally, since MR-MAPs are easier to prepare and administer, their use could significantly lessen the human resource training needs. Such an approach was considered particularly beneficial for targeting high-risk groups, facilitating outreach, initiating school-based immunizations, responding to outbreaks, addressing natural disasters, conducting mop-up campaigns, and strengthening case surveillance. Ensuring the success of these use cases would also hinge on providing these workers with proper training and consistent supervision.

The ability for the *self-administration of MR-MAPs with or without health worker or community health worker assistance* offers promising prospects, but their implementation poses notable challenges. The potential for caregivers to administer the vaccines themselves can promote a sense of responsibility towards personal health and possibly diminish anxieties associated with vaccines. These use cases could be especially beneficial during mass vaccination campaigns. However, it was highlighted that several factors need addressing before such use cases can be broadly adopted. Concerns were raised around the documentation of vaccination coverage and ensuring compliance, as well as the monitoring of AEFIs caused mainly by programmatic errors. Similar concerns were shared by stakeholders who contributed to the development of the initial use cases. Moreover, operating procedures for waste management and proper disposal need to be explored. While stakeholders did not rule out these use cases, they noted that significant evidence on the feasibility needs to be generated that would support potential policy and programmatic changes to enable such use.

#### 3.3.1. The Ability of Community Health Workers (CHWs) to Deliver Vaccines

As discussed above, workshop participants confirmed the desirability of Community Health Workers (CHWs) to deliver MR vaccines using MAPs which could potentially extend vaccination to underserved populations and alleviate barriers around the shortage of health workers. However, significant policy and programmatic changes are needed to allow for the uptake through these use cases.

CHWs typically reside within the communities they serve, often receiving lower levels of formal education and clinical training than professional healthcare personnel, specifically nurses and doctors. They are either paid community health workers, or volunteers, and they play a key role in supporting and extending health care services to vulnerable and difficult-to-reach populations. Their role in administering vaccines varies across countries and some national policies prevent CHWs from delivering vaccines [20]. From the countries participating in the workshops, some could be categorized as more permissive for the CHW delivery of vaccines (Ethiopia), those that permit vaccine delivery with CHWs in special situations, such as hard-to-reach populations or conflict zones (Indonesia, Uganda), and those that do not permit vaccine delivery with CHWs (Jamaica).

Thus, policy change is needed to allow CHWs to administer vaccines in certain countries. Participants from all countries, except Jamaica, expressed an openness to revising their policies, especially given the MR-MAP is a non-injectable vaccine. Second, a programmatic change is needed to further formalize the role of CHWs and ensure adequate financial compensation for their extended services. The opinions between participants differed about the feasibility of including the CHWs’ salaries in the budget, and they highlighted that this would require additional advocacy.

#### 3.3.2. Implications of Integrating MR-MAPs Delivery with Other Non-MAP Vaccines

In Uganda, as well as in several other countries, the potential co-administration of MR-MAPs with other non-MAP vaccines during routine immunization has been discussed. Currently, the MR vaccine is co-administered with yellow fever vaccine in some countries, and there is potential to co-administer it with typhoid, malaria, and other vaccines. However, there is a concern that the unique advantages of MR-MAPs might be reduced when administered alongside other vaccines. For instance, the significant benefit of MR-MAPs’ increased thermostability might not be fully appreciated if the cold chain during outreach or campaigns must be maintained for other co-delivered vaccines. Conversely, co-administering MR-MAPs with other vaccines or health interventions could introduce operational efficiencies, such as freeing up cold chain space for the last mile of vaccine delivery or optimizing session time. Importantly, key MR-MAP attributes such as ease of preparation and administration, and non-injectable delivery, were perceived as sufficient advantages for the immunization program to justify MR-MAP use even without enjoying the full advantages.

### 3.4. Step 4: Understanding Decision Pathways and Criteria for Decision Making About the Introduction of MR-MAPs in the Immunization Programme

Understanding the criteria for decision making about the introduction of MR-MAPs in countries and the evidence needs to inform that process is important for the optimal design of phase III clinical trials, identification of key implementation research, development of financing plans, and introduction and communication strategies.

Countries expressed that the typical decision-making process is expected to be employed to determine whether to introduce and integrate an MR-MAP. With political interest and commitment to measles and rubella elimination, Ministries of Health would likely request a recommendation from National Immunization Technical Advisory Groups (NITAGs) about if and how to introduce MR-MAPs.

As part of the discussion, countries deliberated on a hypothetical potential full switch from MR needle and syringe vaccines to MR-MAPs or a partial switch designated only for certain hard-to-reach populations or limited to delivery strategies such as campaigns. The discussion included the assumption that MR-MAPs will be more expensive than N/S vaccines; however, with growing demand, the price of MAPs should decrease, hopefully making the innovation financially sustainable for countries. Stakeholders identified a wide range of decision criteria, beyond just the cost of the product, such as impact on coverage and equity, waste management implications, and measles elimination targets that would be considered during decision making. Budget impact, cold chain requirements, and vaccine acceptability also featured prominently in countries’ considerations. A consolidated list of the evidence required to consider the introduction of MR-MAPs by the participating countries is available in the Supplementary Material, Table S2. It was further suggested that adoption of MR-MAPs would be unlikely if MR-MAPs are not prequalified by WHO.

Ahead of broad-scale implementation, the preference of the nine participating countries leant towards generating local evidence. This might take the form of feasibility studies aimed at addressing vital implementation questions, pilot projects, or subsequently, phased introductions.

#### 3.4.1. Understanding the Feasibility of Mixed Delivery

The feasibility of implementing mixed delivery, where MR vaccines are delivered via MAPs in certain sub-national settings or delivery strategies and through needle and syringe in others, would be influenced by an array of factors that span policy, programmatic, and procurement domains. Countries highlighted that lessons learned from the switch from 10 to 5 dose vials of measles containing vaccine (MCV) should be leveraged to inform the mixed use of MR vaccines delivered by N/S and MAP.

A standard pathway to introduce a new vaccine into a country immunization programme is expected to be followed for MR-MAPs. In addition, inclusion of MAPs into an immunization programme for delivery alongside N/S would also necessitate guidance from technical agencies (e.g., WHO), development of standard operating procedures, and financial backing.

From a programmatic perspective, there are training requirements to facilitate the delivery of mixed presentations of vaccines. Workshop participants expressed that particular attention should be given to training focusing on handling different thermostability profiles, administration routes, and vaccine presentations. Appropriate training would need to be developed and implemented to enable safe and effective administration of MR-MAPs by CHWs—if countries’ policies permit. Training and guidance to inform and sensitize communities about MR-MAPs and prevent concerns about different presentations of MR vaccines would be required. Additional training and guidance priorities that were expressed by participating countries included assessment of cold chain capacity coupled with appropriate microplanning for campaigns and outreach immunization to reduce vaccine wastage. Furthermore, effective waste management protocols and updates to the existing information systems are imperative to accommodate mixed delivery, as the waste disposal procedures for the two presentations might differ.

The feasibility of mixed delivery is also underpinned by supply chain considerations. For countries procuring through UNICEF, it is important to understand whether differentiated supply chains are feasible. Additionally, factors such as supply availability, cost per dose, and minimum order requirements might affect the country decision for a mixed-delivery strategy.

#### 3.4.2. The Impact of Cost on the Decision to Introduce MR-MAPs

The price of MR-MAPs is central to considerations for the introduction of MR-MAPs. While the precise price of MR-MAPs remains uncertain, it is anticipated that they will be more expensive than MR vaccines delivered by N/S. During the workshop in Uganda, different cost scenarios were discussed by stakeholders to identify criteria and incremental impact that would justify the increased price. The discussion was conducted under the assumption that the immunogenicity and safety of MR-MAPs will be non-inferior to N/S delivery.

A substantial price disparity between MR-MAPs and conventional MR vaccines could significantly hinder their inclusion in vaccine programmes. Crucially, before considering broader introduction, countries emphasized the importance of undertaking cost–benefit analyses in pilot evaluations, and considering the results in a broader context of the financial challenges and limited budgets facing countries. Nuanced perspectives emerged from different countries. For instance, participants from Uganda underscored the necessity of a comprehensive evaluation of immunization costs. They believe it is essential to consider costs of MR-MAP delivery and introduction in relation to MR-MAPs’ impact on wastage, health worker time, and the consequences of inaction, such as the continued prevalence of measles and rubella cases, deaths, and cost of outbreak response. Likewise, in the Caribbean and Guyana, there was a strong regional commitment towards MR elimination. The consensus was that if MR-MAPs could more effectively contribute towards and maintain MR elimination, they should be considered, even if the price were to be higher. However, middle-income countries procuring through the PAHO Revolving Fund have stressed the importance of securing fair pricing for MR-MAPs. In Indonesia, the position was more pragmatic; increased cost would be considered as long as it remained within the pre-determined immunization budget. Overall, gaining a comprehensive understanding of the broader impacts and benefits of MR-MAPs is essential for countries to make informed decisions about their potential adoption.

#### 3.4.3. Considerations for Countries Using MMR in Routine Immunization

The introduction of MR-MAPs in regions where Measles, Mumps, and Rubella (MMR) vaccines are in use, such as the American Region, comes with its own challenges and advantages. Although participating PAHO countries acknowledged the benefits of MR-MAPs, they would be unlikely to replace an MMR vaccine for routine immunization with one effective only against MR, losing the mumps protection. Still, participants expressed that the high cost associated with maintaining measles elimination makes MR-MAPs attractive for strategies other than routine immunization, including follow-up campaigns, mop-up activities especially targeting older groups, outbreak responses, and reaching difficult areas. However, if supplementary immunization activities were to be phased out, the comparative advantage of MR-MAPs will likely diminish. Future deliberations in this context should focus on the investment case for an MMR-MAP.

#### 3.4.4. Evidence Generation and Pilot Programmes Ahead of Wide MR-MAP Implementation

Stakeholders in the workshops acknowledged the need for evidence generation ahead of the introduction of MR-MAPs. Indonesia, Ethiopia, Uganda, and Guyana were interested in participating in clinical trials, conducting feasibility or pilot studies to answer outstanding MR-MAP questions and assumptions and contribute to global evidence generation.

In addition, the majority of countries said that successful implementation of MR-MAPs is likely to require introduction pilots in areas with well-functioning immunization programmes before a wider introduction. These pilot programmes serve a dual purpose: First, they aim to test key assumptions associated with the implementation of MR-MAPs, such as their impact on the cold chain, and impact on vaccine wastage, potential budgetary implications, the quality and effectiveness of health worker training, the acceptability by caregivers and the capability of community health workers to effectively administer MAPs. Second, they are an avenue to curate communication and training materials anchored in local data and perspectives. Engaging local community and religious leaders in this process ensures that the introduction of MR-MAPs resonates with the community and facilitates a smoother, broader rollout in the rest of the country.

## 4. Recommendations

The below recommendations, based on the findings from these workshops, should not supersede those found in the WHO/UNICEF MR-MAP TPP and are intended to complement or highlight attributes that need particular focus during the product development, on the basis of country stakeholder feedback.

On MR-MAP attributes, the following recommendations can be made:

- **Easy Preparation and Administration**: Ensure that the MR-MAP operation is intuitive and straightforward, minimizing requirements for training, as well as steps for vaccine preparation and administration, thereby simplifying the process and minimizing potential errors or challenges in the field. Accompany the product with clear, easy-to-understand instructions, potentially augmented by visual aids or digital tutorials, ensuring that even those with minimal training can administer the vaccine effectively.
- **Thermostability Compliant with VVM14 and CTC Indications**: Dedicate efforts to ensure the MR-MAPs meet at least the VVM14, and ideally VVM30 targets, and are compatible with CTC indications for as long a duration as the stability permits, as outlined in the MR-MAP TPP. Ensure sufficient cold chain capacity to accommodate increased volume for MR-MAPs, if needed.
- **Fewer Components**: Strive for a minimalist design that reduces the volume of a MAP, and the number of ancillary devices (including applicators) required for delivering a vaccine dose.
- **Wear Time**: Given the preference for shorter wear time across use cases, developers should aim for MR-MAPs with no higher than one-minute wear time, ensuring the efficacy or thermostability are not compromised. Provide clear information to vaccinators and caregivers about the consequences of wear time of the MR-MAP beyond the stated duration, improving confidence and ensuring safe application during both routine immunization sessions and campaigns.
- **Site of administration**: Evaluate flexibility in the MR-MAP application site given cultural and programmatic challenges and sensitivities. Undertake studies to evaluate the impact of various skin factors and conditions on MR-MAP efficacy, to ensure broad acceptability and generalizability of their use.

On MR-MAP use cases:

- **Study on Caregiver Involvement**: Conduct studies to assess the feasibility and acceptability of having caregivers hold the child’s arm during MR-MAP application, if not already evaluated in trials and if arm restraint is required.
- **Prioritization of Use Cases**: Given the high favourability and potential for broad use of MR-MAPs, should there be financial or supply constraints, prioritize the implementation and scaling of use of MAPs in outreach immunization and campaigns, leveraging community health care workers for effective MR-MAP deployment.
- **Policy and Training**: Develop policies allowing community health workers to administer MR-MAPs and invest in their training.

On MR-MAP introduction and integration, the following recommendations can be made:

- **Research and Feasibility**: In addition to confirmation of safety and immunogenicity of MR-MAPs in clinical trials, conduct operational studies to understand the implementation and integration of MR-MAPs with the immunization programmes in the context of priority use cases.
- **Price Equivalence**: Strive to ensure that the overall cost to immunize a child with MR-MAPs is not significantly higher than MR delivery in multi-dose vials via needle and syringe to encourage widespread adoption.
- **Cold chain assessment**: Conduct comprehensive cold chain impact assessments for MR-MAPs, factoring in volume requirements and thermostability attributes, to inform effective and resource-efficient integration into national immunization programmes.
- **Guidance on CTC**: Develop and support the implementation of clear guidance on CTC for MR-MAPs in countries and regions, as part of the MR-MAP training.
- **Microplanning Tools**: Highlight the need for microplanning tools to account for conditions specific to MR-MAPs, such as CTC, wear time, and way of administering a MAP, to ensure optimized distribution and minimal wastage.
- **Communication**: Prioritize the development of a comprehensive communication strategy to prevent increased hesitancy and concerns stemming from using different vaccine presentations in various populations.
- **Support for pilot studies**: Assist countries expressing interest in MR-MAP pilot programmes to evaluate MAP integration with immunization programmes and measuring impact on MR coverage. Such support should encompass training and communication material development, stakeholder engagement, and evidence generation.

## Data Availability

The original contributions presented in the study are included in the article and the Supplementary Material; further inquiries can be directed to the corresponding authors.

## Supplementary Materials

The following supporting information can be downloaded at: https://www.mdpi.com/article/10.3390/vaccines12091084/s1. Figure S1: List of six use-cases for MR-MAPs identified through previous analyses (7); Table S1: Overview of types of workshop participants per country; Table S2: A list of the identified criteria required by countries to consider the introduction of MR-MAPs.

## References

[1] Minta A.A., Ferrari M., Antoni S., Portnoy A., Sbarra A., Lambert B., Hatcher C., Hsu C.H., Ho L.L., Steulet C. (2023). Progress Toward Measles Elimination—Worldwide, 2000–2022. MMWR Morb. Mortal. Wkly. Rep..

[2] World Health Organization (WHO) (2017). Measles vaccines: WHO position paper April 2017. Wkly. Epidemiol. Rec..

[3] Hasso-Agopsowicz M., Crowcroft N., Biellik R., Gregory C.J., Menozzi-Arnaud M., Amorij J.-P., Gilbert P.A., Earle K., Frivold C., Jarrahian C. (2022). Accelerating the Development of Measles and Rubella Microarray Patches to Eliminate Measles and Rubella: Recent Progress, Remaining Challenges. Front. Public Health.

[4] Fu H., Abbas K., Malvolti S., Gregory C., Ko M., Amorij J.-P., Jit M. (2023). Impact and cost-effectiveness of measles vaccination through microarray patches in 70 low-income and middle-income countries: Mathematical modelling and early-stage economic evaluation. BMJ Glob. Health.

[5] Gavi (2020). The Vaccine Innovation Prioritisation Strategy (VIPS). https://www.gavi.org/our-alliance/market-shaping/vaccine-innovation-prioritisation-strategy.

[6] World Health Organization (WHO) (2019). Measles-Rubella Microarray Patch (MR-MAP) Target Product Profile. https://www.who.int/publications/i/item/9789240000209.

[7] Malvolti S., Ko M., Menozzi-Arnaud M., Mantel C., Jarrahian C., Amorij J.P., Giersing B., Hasso-Agopsowicz M. (2023). Exploring potential applications of measles and rubella microarray patches (MR-MAPs): Use case identification. Front. Public Health.

[8] Guillermet E., Alfa D.A., Phuong Mai L.T., Subedi M., Demolis R., Giersing B., Jaillard P. (2019). End-user acceptability study of the nanopatch^TM^; a microarray patch (MAP) for child immunization in low and middle-income countries. Vaccine.

[9] PATH (2019). The PATH Center of Excellence for Microarray Patch Technology. https://www.path.org/our-impact/resources/path-center-excellence-microarray-patch-technology/.

[10] Baker B., Bermingham I.M., Leelasena I., Hickling J., Young P.R., Muller D.A., Forster A.H. (2023). Safety, Tolerability, and Immunogenicity of Measles and Rubella Vaccine Delivered with a High-Density Microarray Patch: Results from a Randomized, Partially Double-Blinded, Placebo-Controlled Phase I Clinical Trial. Vaccines.

[11] Joyce J.C., Collins M.L., Rota P.A., Prausnitz M.R. (2021). Thermostability of Measles and Rubella Vaccines in a Microneedle Patch. Adv. Ther..

[12] Ko M., Malvolti S., Cherian T., Mantel C., Biellik R., Jarrahian C., Menozzi-Arnaud M., Amorij J.P., Christiansen H., Papania M.J. (2022). Estimating the future global dose demand for measles-rubella microarray patches. Front. Public Health.

[13] Adigweme I., Yisa M., Ooko M., Akpalu E., Bruce A., Donkor S., Jarju L.B., Danso B., Mendy A., Jeffries D. (2024). A measles and rubella vaccine microneedle patch in The Gambia: A phase 1/2, double-blind, double-dummy, randomised, active-controlled, age de-escalation trial. Lancet.

[14] Scarnà T., Menozzi-Arnaud M., Friede M., DeMarco K., Plopper G., Hamer M., Chakrabarti A., Gilbert P.A., Jarrahian C., Mistilis J. (2023). Accelerating the development of vaccine microarray patches for epidemic response and equitable immunization coverage requires investment in microarray patch manufacturing facilities. Expert Opin. Drug Deliv..

[15] Forster A., Junger M. (2022). Opportunities and challenges for commercializing microarray patches for vaccination from a MAP developer’s perspective. Hum. Vaccines Immunother..

[16] Giersing B., Shah N., Kristensen D., Amorij J.-P., Kahn A.-L., Gandrup-Marino K., Jarrahian C., Zehrung D., Menozzi-Arnaud M. (2021). Strategies for vaccine-product innovation: Creating an enabling environment for product development to uptake in low- and middle-income countries. Vaccine.

[17] TechNet-21 (2023). Tools and Platforms that Support Selection, Access, and Service Strategies (17th TechNet Conference). https://www.youtube.com/watch?v=hsdQfBbVtLk.

[18] World Health Organization (WHO) (2021). Guide for Conducting a Situation Analysis of Immunization Programme Performance. https://www.who.int/publications/m/item/guide-and-workbook-for-conducting-a-situation-analysis-of-immunization-programme-performance.

[19] World Health Organization (2020). CAPACITI Decision Support Tool Manual. https://www.who.int/publications/m/item/capaciti-decision-support-tool-manual.

[20] Gibson E., Zameer M., Alban R., Kouwanou L.M. (2023). Community Health Workers as Vaccinators: A Rapid Review of the Global Landscape, 2000–2021. Glob. Health Sci. Pract..

