# Supplementary material for: Exploring Important Attributes, the Potential Use Cases and Feasibility of Introduction of Measles and Rubella Microarray Patches (MR-MAPs): Insights from Nine Countries
Source: Vaccines (Basel). 2024 Sep 23;12(9):1084. doi: 10.3390/vaccines12091084 (PMC11435686; doi:10.3390/vaccines12091084)
Supplement: Supplementary file 1 [file vaccines-12-01084-s001.zip › vaccines-3135262-supplementary.pdf]

Supplementary material

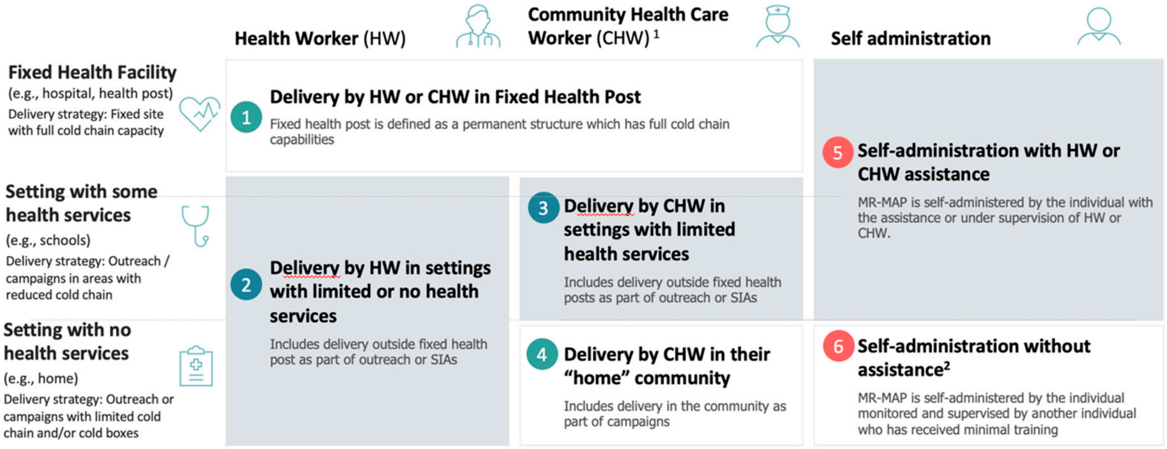

Figure S1. List of six use-cases for MR-MAPs identified through previous analyses(7).

WORKSHOP	DRIVERS OF COUNTRY SELECTION	DATE	PARTICIPANTS
Ethiopia (AFRO)	• IA2030 priority country, top 20 zero-dose children<br>• Discussions building on current evaluations for switch in MCV presentation<br>• Prior engagement with the CAPACITI tools on decision question relating to MCV	August 2022	**44 participants from**: Ministry of Health, district health officers, Institute for Public Health, National Immunization Technical Advisory Group (NITAG), Academia, Ethiopian Food and Drug Administration (FDA), CSO representatives (Project Hope), donors (USAID), implementing partners (CHAI, PATH), UNICEF, WHO HQ/RO/CO.
Indonesia (SEARO)	• IA2030 priority country, top 20 zero-dose children<br>• Local production of vaccines<br>• Archipelagic state and each of the islands has unique challenges	Septembe r 2022	**49 participants from:** Ministry of Health, Center for Disease Control Indonesia, NITAG, Indonesia FDA, Pharmaceutical Resilience, UNICEF, WHO HQ/CO
Guyana, Belize, Jamaica, Trinidad and Tobago, Barbados, Suriname (PAHO)	• MIC countries<br>• High MMR coverage, at high cost for sustaining measles elimination<br>• Pooled procurement through Revolving fund<br>• Small populations, close geographic proximity- testing the assumption of archetype countries	May 2023	**54 participants from:** Ministry of Health, NITAG, Interagency Coordination Committee, Nurses, EPI Supervisors, cold chain advisor, vaccine safety, regional commission for measles elimination, Revolving Fund, The Caribbean Public Health Agency (CARPHA), WHO/PAHO
Uganda (AFRO)	• Diverse sub-national contexts<br>• Low measles incidence, but no elimination yet<br>• High refugee population	July 2023	**39 participants from:** Ministry of Health, district health officers, NITAG, PATH, USAID, Center for disease control, research, cold chain, research, and implementation

Table S1. Overview of types of workshop participants per country

A list of prioritised barriers across the seven EPI categories

PROGRAMME MANAGEMENT & FINANCING

- Inadequate subnational budgeting (unavailability and challenges in disbursement of funds)
- Inadequate funding to non-HR related activities to combat vaccine hesitancy

HUMAN RESOURCES MANAGEMENT

- Inadequate supply of health staff
- Suboptimal supervision
- Inadequate training
- Poor staff motivation

VACCINE SUPPLY, QUALITY & LOGISTICS

- Challenges in transport of vaccines to remote delivery
- Sub-optimal waste management
- Inappropriate monitoring of heat or freeze exposure during transport or storage
- Inadequate cold chain (e.g. refrigerators are available but in some parts not functional due to lack of maintenance funds)
- Poor vaccine distribution capacity

SERVICE DELIVERY

- Long distance and travel time pose challenges with service delivery
- Special and remote populations require more outreach efforts/resources
- Inadequate strategies used to reach underserved or under-immunized populations

IMMUNIZATION COVERAGE & AEFI MONITORING

- Lack of functional AEFI surveillance system
- Inadequate reporting & recording tools
- Low quality data linked to incompleteness
- Challenges in determining accurate denominators

DISEASE SURVEILLANCE

- Data is inadequate, not available in timely manner or not used for action

DEMAND GENERATION

- Lack of confidence & trust in vaccines among certain populations
- Fear of multiple injections
- Cultural/religious concerns
- Inadequate communication

CRITERIA	EVIDENCE NEEDED
Impact on coverage and equity	• Data is preferred from pilot in country or countries with similar barriers to immunization. Modeling data useful if based on local data.
Budget Impact	• Different use scenarios at sub-national level and national level for budget impact.
Cold chain capacity	• Volume needs and cold chain availability assessment
Local production	• Possibility of tech-transfer as per national strategy
Containing haram ingredients	• Should not contain haram ingredients as there will be community resistance
Waste management	• Impact assessment on waste management needs, including toxicity and biohazard scenarios
Safety (AEFI)	• Post market surveillance data from other countries
Community acceptance	• BeSD survey to understand community acceptance in different areas, key informant interviews, social media review, religious leaders acceptance
Cost-effectiveness	• Cost-benefit analysis, cost comparisons from piloting in country or other countries within the region

Table S2. A list of the identified criteria required by countries to consider the introduction of MR-MAPs
